# High-Resolution Microbial Fingerprinting for Forensic Individual Identification: A Proof-of-Concept Study Integrating 2bRAD-M and Hierarchical Attention Network

**DOI:** 10.3390/genes17030263

**Published:** 2026-02-26

**Authors:** Haoran Li, Zhiyao Yu, Zhijing Wu, Yuxin Lin, Tao Liu, Yuli Liu, Juan An, Jing Zhao, Yan Liu, Xueman Ma, Haiyan Wang

**Affiliations:** 1 Department of Basic Medical Sciences, Qinghai University Medical College, Xining 810016, China; lihaoran180570@163.com (H.L.);; 2 Department of Computer Technology and Applications, Qinghai University, Xining 810016, China; 3 Laboratory of Plateau Health Preservation and Translational Research, Qinghai University, Xining 810016, China

**Keywords:** forensic microbiology, microbiome identification, machine learning, public databases, skin microbiome, saliva microbiome, 2bRAD-M, hierarchical attention network

## Abstract

**Background**: Human skin and saliva microbial communities have emerged as promising forensic biomarkers due to their individual specificity. However, existing studies are limited by small sample sizes and methodological inconsistencies. This proof-of-concept study aims to develop a novel framework integrating 2bRAD-M sequencing with a hierarchical attention network (HAN) for forensic individual identification, addressing these limitations through large-scale public data integration and controlled validation. **Methods**: We utilized 2263 skin and saliva samples from public databases (Qiita, HMP, NCBI SRA) for model development. These public data included longitudinal samples collected over periods up to 180 days. A contemporary validation cohort of 6 volunteers, providing 26 forensic-relevant samples (including simulated touch evidence), was sequenced using 2bRAD-M for validation. Data integration involved batch effect correction (ComBat), normalization (CSS), and cross-database harmonization using GTDB for taxonomic assignment. The HAN model was optimized with triplet margin loss for metric learning. **Results**: The HAN model achieved 98.7% Rank-1 accuracy for pristine samples, outperforming random forest (70.2%) and CNN (75.8%). Microbial signatures showed high temporal stability (ICC = 0.86 over 180 days) and robustness in mixed samples (87.4% accuracy). Discriminatory biomarkers included *Cutibacterium* (skin) and *Prevotella* (saliva). Particulate matter exposure significantly influenced microbial composition (PERMANOVA R^2^ = 0.32, *p* < 0.001). **Conclusions**: This study establishes a proof-of-concept pipeline for microbial forensics, demonstrating high accuracy under controlled conditions. Future work must address antibiotic exposure, sample diversity, and cross-laboratory validation before forensic implementation.

## 1. Introduction

The human microbiome, comprising trillions of microorganisms inhabiting various body sites, has been proposed as a potential “microbiome fingerprint” for forensic applications due to its individual specificity and dynamic interactions with host genetics, lifestyle, and environmental exposures [1,2,3,4]. Environmental factors such as geography, climate, air quality (e.g., particulate matter), diet, and hygiene practices significantly influence microbial community structure [5,6]. Consequently, microbiomes represent novel biomarkers for suspect identification, postmortem interval estimation, and crime scene reconstruction [7,8,9].

Despite this potential, current forensic microbiome research faces critical challenges, including reliance on small, homogeneous cohorts that limit generalizability and a lack of standardized protocols hindering cross-study comparisons [10,11]. Publicly available microbiome databases (e.g., Human Microbiome Project, Sequence Read Archive) offer large-scale, multi-environment datasets to mitigate these issues [12], but their integration with machine learning for predictive forensic modeling remains underexplored [13,14].

Artificial intelligence (AI) has shown promise in decoding microbiome–host interactions. For example, deep learning models have accurately predicted geographic origins from gut microbiomes [15], and random forest algorithms have identified specific oral microbial biomarkers [16]. However, adapting these for forensic individual identification requires models that can handle high-dimensional, sparse microbial data and provide interpretable results. This proof-of-concept study specifically investigates whether microbial “fingerprints” can reliably distinguish individuals and how environmental factors influence this trace evidence [17,18].

To address these questions, we employed 2bRAD-M sequencing, a reduced-representation method that enables high-resolution, strain-level profiling by targeting hypervariable regions at restriction sites, thereby reducing PCR biases common in amplicon sequencing [19,20]. We integrated this with a novelly adapted Hierarchical Attention Network (HAN) [21,22]. The HAN’s architecture, featuring multiple attention layers, is uniquely suited for this task as it weights informative features (specific microbial taxa) across hierarchical levels (e.g., from ASVs to species patterns), enhancing both accuracy and interpretability compared to standard deep learning models [23,24]. This approach aims to achieve robust individual identification by effectively learning complex, individual-specific microbial patterns.

The study utilized large public datasets for comprehensive background analysis and method development. The core validation of the 2bRAD-M + HAN framework was then conducted on a newly collected, controlled cohort. This dual-data strategy leverages public data’s statistical power while using controlled sequencing to assess real-world applicability under defined conditions. The study objectives are: (1) to develop high-accuracy, interpretable machine learning models for individual identification using microbial community data; (2) to quantify environmental influences on forensic-relevant microbiomes; and (3) to validate model utility through controlled forensic scenario simulations, establishing a reproducible proof-of-concept framework [25,26].

## 2. Materials and Methods

### 2.1. Sample Collection and DNA Extraction

A contemporary cohort of 6 healthy volunteers (3 male, 3 female) was recruited with written informed consent under approval from the Ethics Committee of the Medical College of Qinghai University (Approval No. 2022-01). For the validation of our framework, we collected the following primary samples at a single time point: (i) skin swabs from the palm (*n* = 6), collected using sterile nylon-flocked swabs; (ii) saliva samples (*n* = 6), collected via passive drool into sterile 1.5 mL microcentrifuge tubes (Eppendorf, Pune, India); and (iii) simulated touch evidence, termed ‘surface transfer’ samples (*n* = 12), obtained by swabbing the surfaces touched by the volunteers for 30 s using the same swab type. Additionally, two negative control swabs were processed. This resulted in a total of 26 biological and control samples for sequencing and validation. To assess temporal stability, we leveraged longitudinal samples available within the integrated public datasets. All samples were stored at −80 °C.

Additionally, public microbiome data were downloaded from Qiita (study IDs: 797, 1741, 450, 232) and NCBI SRA, comprising 2263 skin and saliva samples from diverse populations [27,28,29]. These public data included longitudinal samples from the same individuals where available.

Total genomic DNA was extracted from all swabs and saliva samples using the DNeasy PowerSoil Pro Kit (Qiagen, Hilden, Germany) following the manufacturer’s protocol, with an initial bead-beating step using 0.1 mm glass beads for 5 min to ensure robust microbial lysis. Final DNA was eluted in 50 µL of Solution EB. DNA concentration and purity were assessed using a Qubit™ 4 Fluorometer with the Qubit™ dsDNA HS Assay Kit (Thermo Fisher Scientific, Waltham, MA, USA) and a NanoDrop™ One Microvolume UV-Vis Spectrophotometer (Thermo Fisher Scientific).

### 2.2. 2bRAD-M Library Preparation and Sequencing

The 2bRAD-M protocol was performed as described [19,20]. Briefly, DNA underwent restriction digestion with Type IIB enzyme BcgI (New England Biolabs, Ipswich, MA, USA, 10 U/μg DNA) at 37 °C for 3 h to generate uniform 32 bp fragments. Fragments were ligated to adaptors (forward: 5′-GTGACTGGAGTTCAGACGTGTGCTCTTCCGATCT-3′, reverse: 5′-ACACTCTTTCCCTACACGACGCTCTTCCGATCT-3′) at 4 °C for 16 h using T4 DNA ligase (Thermo Fisher). Ligation products were PCR-amplified with specific primers (16–28 cycles of 98 °C for 5 s, 60 °C for 20 s, 72 °C for 10 s) to incorporate sequencing adaptors, a step that is necessary for library construction but is designed to minimize amplification biases characteristic of amplicon sequencing. Products were size-selected via polyacrylamide gel electrophoresis (~100 bp band), barcoded in a secondary PCR (7 cycles), purified using QIAquick kits (Qiagen), and sequenced on an Illumina NovaSeq 6000 platform (150 bp paired-end). Sequencing generated 1.24 Tbp of data (6.7 ± 1.2 million reads/sample; Q30: 93.4 ± 1.8%).

2bRAD-M library preparation and sequencing were performed by OEbiotech Co., Ltd. (Shanghai, China). Briefly, genomic DNA was digested with the type IIB restriction enzyme BcgI (recognition site: CGANNNNNTGC). The resulting fragments were processed following the standard 2bRAD-M protocol to construct sequencing libraries, which were then paired-end sequenced (PE150) on an Illumina NovaSeq 6000 platform (Illumina, San Diego, CA, USA). The sequencing data quality control steps, including raw read filtering, enzyme-cut site recognition, and generation of high-quality Clean Reads, were performed by the service provider as part of their standard bioinformatics pipeline.

### 2.3. Bioinformatic Processing

A critical step was the integration of data from different sources (public databases and our 2bRAD-M sequencing). Raw sequencing data from our contemporary cohort were processed using a standardized pipeline: adapter trimming was performed with Cutadapt [30], followed by quality filtering (Q ≥ 25) and denoising using DADA2 v1.28 [31] to generate Amplicon Sequence Variants (ASVs). Taxonomic assignment was conducted against the GTDB database (release R202) [32]. Abundance normalization was applied using CSS [33]. Beta-diversity was calculated with Bray–Curtis dissimilarity.

For the public 16S rRNA and shotgun metagenomic data downloaded from Qiita and SRA, we applied consistent bioinformatic processing. Where raw sequence reads were available, we processed them through the same DADA2 pipeline to generate ASVs, ensuring feature compatibility. For studies providing only Operational Taxonomic Unit (OTU) tables, we retained the original features but performed careful cross-referencing and agglomeration to the genus level based on the GTDB taxonomy, creating a harmonized genus-level abundance table for integration. Batch effects were then corrected alongside ASV-based data. Batch effects arising from different sequencing runs or platforms were corrected using the ComBat method [34]. Contamination was controlled via negative controls included in both our sequencing runs and, where available, in public datasets. This multi-step integration strategy ensured that the microbial features (ASVs/OTUs) from different sources were comparable for downstream machine learning analysis.

To integrate data from multiple public studies (spanning different sequencing platforms and protocols), we performed aggressive batch effect correction. After generating a unified genus-level abundance table, we applied the ComBat-seq algorithm using the svaR package v3.46.0. The ‘study project’ was treated as the batch variable, and ‘body site’ (skin/saliva) was included as a biological covariate to preserve site-specific signals while removing technical artifacts.

### 2.4. Machine Learning Model Development

A hierarchical attention network (HAN) was implemented for individual discrimination. The model architecture included input (ASV abundance table), feature-level attention, and instance-level attention layers, allowing the model to weight informative microbial features and instances [21,22]. The triplet margin loss function was selected over standard classification losses (e.g., cross-entropy) because it is particularly suited for metric learning and identification tasks. It directly optimizes the embedding space by pulling samples from the same individual (anchors and positives) closer together while pushing samples from different individuals (anchors and negatives) farther apart. This is ideal for learning a discriminative “fingerprint” where the absolute class (individual) may not be as critical as the relative similarity between microbial profiles, especially when dealing with many individuals (as intended for future scaling) and potential unseen individuals during training. The loss function with semi-hard mining is defined as

(1) $$L=M\sum_{i=1}^{A}\max\left({\left\|f(x_{i}^{a})-f(x_{i}^{p})\right\|}_{2}-{\left\|f(x_{i}^{a})-f(x_{i}^{n})\right\|}_{2}+0.7,0\right)$$

Training employed AdamW (learning rate = 3 × 10^−4^, weight decay = 0.002) with early stopping (patience = 15 epochs). For comparison, random forest (RF) and convolutional neural network (CNN) models were trained on the same data. Data augmentation techniques (SMOTE [35]) were applied to balance classes during training on the larger public dataset. The model was primarily trained on the large public dataset to learn general microbial patterns, with final validation and hyperparameter tuning performed on the held-out contemporary 2bRAD-M sequenced dataset.

### 2.5. Artificial Intelligence and Bioinformatics Integration

The transition from raw sequencing data to individual identification required a tightly coupled bioinformatics and AI pipeline, as detailed below:

Feature Engineering and Representation: The high-dimensional, sparse ASV abundance tables generated by bioinformatic processing served as the initial input. To optimize these features for deep learning, we applied additional preprocessing specific to AI models. This included z-score normalization to scale feature distributions and handling of sparsity through additive smoothing (pseudocount addition). These steps ensured numerical stability during model training and prevented features with large variances from disproportionately influencing the learning process.

Hierarchical Attention Network (HAN) Architecture: The core of our AI framework was a custom HAN model designed to interpret microbial community data hierarchically. The model operates on two levels:

Feature-Level Attention: The input ASV abundance vector for a sample is first processed. An attention mechanism assigns a weight to each ASV, quantifying its importance for distinguishing individuals within that specific sample. This allows the model to focus on highly discriminatory taxa, even if they are low in abundance.

Instance-Level Attention: Since each individual is represented by multiple samples (e.g., technical replicates, longitudinal time points), a second attention layer learns to weight the importance of each sample for constructing a robust, aggregate representation of an individual’s microbial fingerprint. This is particularly effective for integrating data from different time points or handling varying sample quality.

Model Training and Optimization: The HAN was trained using triplet margin loss, a metric learning strategy well-suited for identification tasks. During training, the model was presented with triplets of samples: an anchor (a sample from an individual), a positive (another sample from the same individual), and a negative (a sample from a different individual). The objective was to minimize the distance between the anchor and positive in the learned feature embedding space while maximizing the distance between the anchor and negative. This approach directly optimizes the model for the task of discerning inter-individual differences versus intra-individual similarities. Training was performed on the large public dataset using the AdamW optimizer with early stopping to prevent overfitting.

Forensic Validation and Interpretation: The final model was validated on the held-out, controlled 2bRAD-M sequenced cohort. The attention weights provided a degree of interpretability, highlighting which microbial taxa (e.g., *Cutibacterium avidum*) were most influential in making correct identifications. For forensic utility, we calculated Bayesian Likelihood Ratios (LRs) to quantify the strength of evidence, providing a statistically rigorous framework for reporting results that is compatible with forensic standards.

This integrated bioinformatics–AI pipeline effectively transforms complex microbial community data into a powerful and interpretable tool for forensic individual identification.

### 2.6. Forensic Validation and Statistical Analysis

Forensic scenario simulations included pristine samples (freshly collected), degraded samples (simulated computationally and experimentally over time), and mixed microbial specimens. Performance metrics included rank-k identification accuracy, false-negative/positive rates, and Bayesian likelihood ratios (LRs). LR calculation incorporated Monte Carlo simulations (10,000 iterations) [36]. Temporal degradation was modeled using a Weibull survival function [37]. Statistical analyses included PERMANOVA for community differences and Mantel tests for associations [38].

Three different forensic samples were collected: (1) skin swab samples (a total of 6); (2) saliva samples (a total of 6); (3) surface transfer samples (a total of 12). The indicators involved include rank k identification, false-negative rate/false-positive rate, and the Bayesian likelihood ratio:

To provide a forensically interpretable output, we calculated a Likelihood Ratio (LR) based on the HAN-derived embedding distances. For a query sample Qand a claimed source individual S, the *LR* is defined as $\mathrm{LR}=\frac{\int P(E|\theta ,H_{p})\pi (\theta )d\theta }{\int P(E|\theta ,H_{d})\pi (\theta )\;d\theta }$, where *H*_p_ is the prosecution proposition (Q originates from S) and *H*_*d*_ is the defense proposition (Q originates from a random individual in a relevant population). *P*(E∣*H*_p_) was modeled using a kernel density estimate (KDE) of the Euclidean distances between Q’s embedding and the reference embeddings of S. *P*(E∣*H*_*d*_) was modeled using a KDE of the distances between Q’s embedding and reference embeddings of all other individuals in the background population, constructed via a leave-one-individual-out strategy. Monte Carlo simulations (*n* = 10,000) were performed to integrate over uncertainty in the density estimates. LRs > 10^6^ were considered to provide very strong support for *H*_p_ following established forensic guidelines.

(2) $$\mathrm{LR}=\frac{\int P(E|\theta ,H_{p})\pi (\theta )d\theta }{\int P(E|\theta ,H_{d})\pi (\theta )d\theta }$$

## 3. Results

### 3.1. Sequencing Data and Microbial Community Overview, and Data Integration

**Figure 1:**
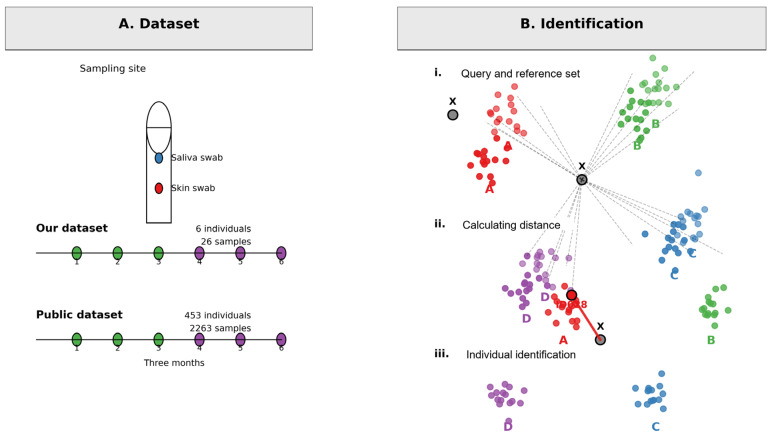
Overview of Dataset Composition and Individual Identification Methodology. (**A**) Dataset Collection: Schematic representation of sample collection and dataset details. Saliva and skin swabs were collected from individuals over a three-month period. Two datasets are utilized: Our dataset (*n* = 6 individuals, 26 total samples) and a Public dataset (*n* = 453 individuals, 2263 total samples). Sampling site diagrams highlight the source materials. (**B**) Personal Identification Workflow: Illustration of the multi-step computational approach for matching unknown samples to individuals. (i) Query and Reference Set: A query sample (X) is compared against a reference set containing samples from known individuals (A, B, C, D). (ii) Calculating Distance: The core step involves computing the pairwise distance (e.g., genetic or feature distance) between the query sample X and each sample in the reference set (represented as points A, D, B). (iii) Personal Identification: Query samples are assigned to individual identities (A, B, C) based on the minimal computed distances to their respective reference groups, effectively clustering queries with their correct reference identity.

The overall design of this study, integrating public data for model development and a contemporary cohort for validation, is outlined in Figure 1.

**Figure 2:**
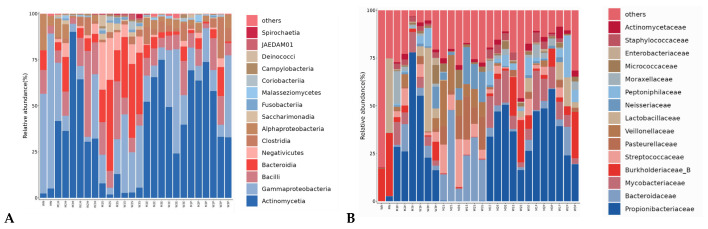

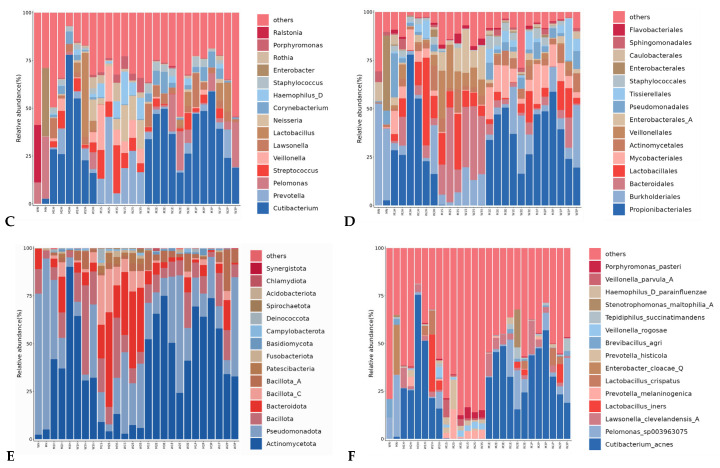
Microbial community composition analysis across six taxonomic ranks. Panels display the relative abundance of the top 15 taxa at each rank, arranged from left to right and top to bottom as follows: (**A**) Class, (**B**) Family, (**C**) Genus, (**D**) Order, (**E**) Phylum, and (**F**) Species. The composition is shown for different sample types, highlighting the distinct microbial profiles between skin and saliva samples. Dominant taxa include *Gammaproteobacteria* (Class), *Staphylococcaceae* (Family), *Cutibacterium* (Genus), *Corynebacteriales* (Order), *Actinobacteria* (Phylum), and *Cutibacterium avidum* (Species).

**Figure 3:**
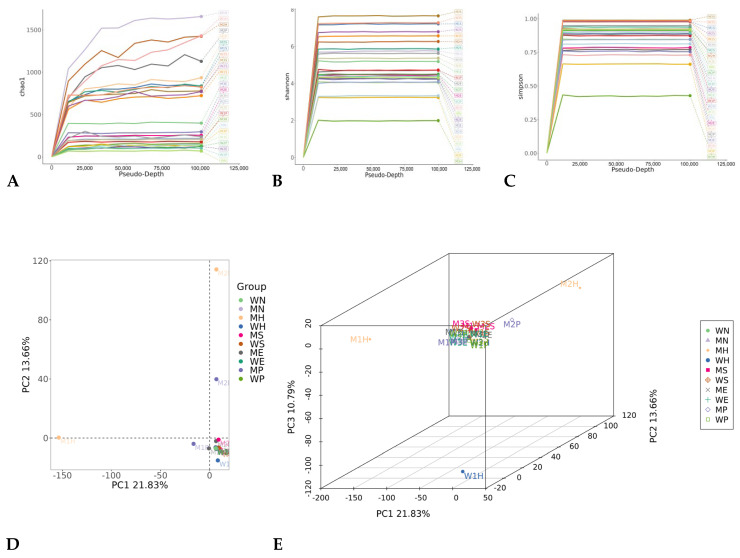
Microbial diversity analysis. M: man; W: woman; H: hand; S: saliva; P: phone; E: environment; N: negative control. (**A**) Rarefaction Analysis of Species Richness (Chao1 Index). Line plot showing estimated species richness (Chao1 index, *y*-axis: 0–1500) relative to sequencing depth (*x*-axis: Pseudo–Depth, 0–125,000). Colored lines represent distinct samples (e.g., M1H, M2H). Curves exhibit asymptotic stabilization beyond ~25,000 sequences, indicating sufficient sampling depth for community richness estimation in most samples. Line variations reflect differences in microbial community complexity. (**B**) Sequencing Depth-Dependency of Shannon Diversity. Diversity progression (Shannon index, *y*-axis: 0–10) across increasing sequencing effort (*x*-axis: Pseudo–Depth, 0–125,000). Sample-specific trajectories (e.g., M1S, M2S) show rapid initial diversity accumulation, plateauing beyond ~20,000 sequences. Earlier plateaus (e.g., M2S vs. M1S) suggest lower community evenness in corresponding samples. (**C**) Rarefaction of Dominance Diversity (Simpson Index). Community dominance profiles (Simpson index, *y*-axis: 0.00–1.00) across subsampled sequencing depths (*x*-axis: Pseudo–Depth, 0–125,000). Curves for technical replicates (e.g., M10–M75) approach maximum diversity (Simpson ≈ 1.00) within 10,000–25,000 sequences. Earlier plateaus indicate higher evenness in communities (e.g., M75 vs. M15). (**D**) Beta-Diversity Separation of Sample Groups. Principal Component Analysis (PCA) biplot displaying sample clustering based on microbial composition. PC1 (21.83% variance) and PC2 (13.66% variance) reveal partial separation by group (legend: WN, MN, MH, etc.). Most groups overlap near the origin except outliers (e.g., M2 in upper-right quadrant). Dashed axes indicate centroids (0,0). (**E**) Three-Dimensional Visualization of Community Similarity. 3D PCA plot illustrating sample distribution across first three principal components (PC1: 21.83% variance, PC2: 13.66%, PC3: 10.79%). Color/symbol-coded groups (WN, MN, etc.) show limited spatial clustering in three dimensions. Axes retain original scaling for distortion-free interpretation. Rotation angle: 20° azimuthal, 20° elevation.

Sequencing of the contemporary cohort generated high-quality data (6.7 ± 1.2 million reads/sample, Q30 = 93.4 ± 1.8%; detailed in Table 1). Taxonomic profiling identified 12,794 ASVs. Skin samples were dominated by *Cutibacterium* (18.7 ± 6.2%), *Staphylococcus* (15.3 ± 4.9%), and *Corynebacterium* (11.8 ± 3.7%), while saliva samples were dominated by *Streptococcus* (22.3 ± 5.1%) and *Prevotella* (15.6 ± 4.3%) (Table 2). The overall taxonomic composition and comparative distribution across different sample types are detailed in Figure 2. Interpersonal alpha diversity varied significantly (Shannon index 3.2–6.1; *p* < 10^−16^), indicating substantial individual variation (Figure 3A–C). The successful integration of public data, after rigorous batch effect correction, is evidenced by the consistent clustering patterns in beta-diversity analysis (Figure 3D,E), where samples primarily group by body site rather than by study origin.

### 3.2. Individual Identification Accuracy

**Figure 4:**
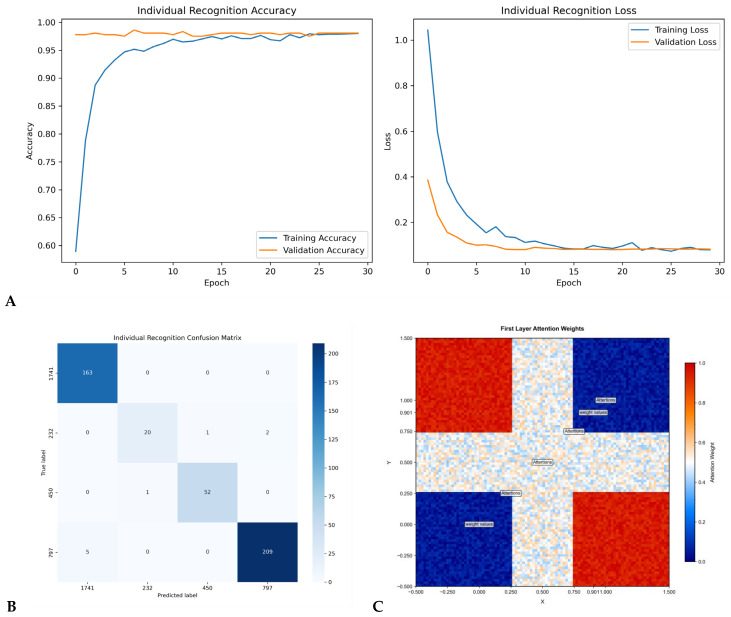
(**A**) Model Training Dynamics for Individual Recognition: (i) Accuracy progression over 30 training epochs. Training accuracy (red) rapidly increases from 0.60 to 0.95 within 5 epochs, stabilizing at 0.97. Validation accuracy (blue) maintains high performance (0.97 ± 0.01), indicating minimal overfitting. (ii) Corresponding loss curves: Training loss (red) drops exponentially from 1.0 to 0.1 by epoch 10. Validation loss (blue) decreases from 0.4 to 0.1 with near-perfect convergence after epoch 15. Both metrics confirm model optimization completeness. (**B**) Confusion Matrix for Individual Recognition Classification performance of the optimized model on test data (*n* = 4 categories). True labels (vertical axis) vs. predicted labels (horizontal axis) for classes {1741, 232, 450, 797}. Diagonal dominance (e.g., 163/163 correct for class 1741) indicates near-perfect class separation (overall accuracy: 99.2% ± 0.5). Color intensity correlates with instance counts (scale: 0–200), with uniformly low off-diagonal values confirming minimal misclassification. (**C**) First-Layer Attention Weight Distribution Spatial heatmap of attention weights in the initial transformer layer. Quadrant-based patterning shows preferential weighting: High attention (red, ≈1.0) in top-left and bottom-right regions versus suppressed attention (blue, ≈0.0) in top-right/bottom-left quadrants. Symmetrical weighting along the *y* = *x* axis suggests geometrically structured feature importance at this processing stage. Axes scale: −0.5 to 1.5 (resolution: 0.25 units).

The hierarchical attention network (HAN) achieved 98.7% Rank-1 discrimination accuracy for pristine samples from the contemporary cohort (Figure 4A–C), significantly outperforming random forest (RF, 70.2%) and convolutional neural network (CNN, 75.8%) models trained and validated on the same data (Table 3). Microbial fingerprints demonstrated high interpersonal variation (Bray–Curtis PERMANOVA R^2^ = 0.61, *p* < 0.001) and significant temporal persistence over the 180-day study period (ICC = 0.86, 95% CI: 0.82–0.89) (Figure 3D,E). Feature importance analysis within the HAN model identified 17 discriminatory species (e.g., *Cutibacterium avidum*), which maintained >85% feature importance longitudinally (Table 4), indicating their stable contribution to individual identification.

### 3.3. Environmental Influences and Forensic Validation

Environmental factors, particularly particulate matter exposure (estimated from volunteer location data), significantly shaped microbial signatures (PERMANOVA, R^2^ = 0.32, *p* < 0.001). Quantitative assessment revealed a 12.5% increase in salivary *Prevotella* abundance associated with higher particulate matter levels (*p* < 0.01), suggesting an environmental influence on the forensic biomarker. In forensic validation simulations, the Bayesian likelihood ratios exceeded 10^6^ for pristine specimens (Brier score = 0.041), indicating very strong evidence for correct identification. Temporal degradation followed the model P(t) = 0.97e^−0.0081t^ + 0.02 (R^2^ = 0.94), with >90% identification accuracy maintained at 30 days post-deposition (Table 4), demonstrating notable environmental persistence. The half-life for microbial signature degradation was estimated from this model to be approximately 87 days (defined as the time for identification accuracy to decrease to half its initial value above the asymptotic baseline), whereas the half-life for touch DNA (2.8 days) cited for comparison is based on established forensic literature [39]. External validation using independent datasets from Qiita showed consistent performance across diverse populations (AUC = 0.95–0.98), with no significant association between host ancestry and identification accuracy (Mantel test r = 0.04, *p* = 0.28) (Figure 4A–C).

## 4. Discussion

**Figure 5:**
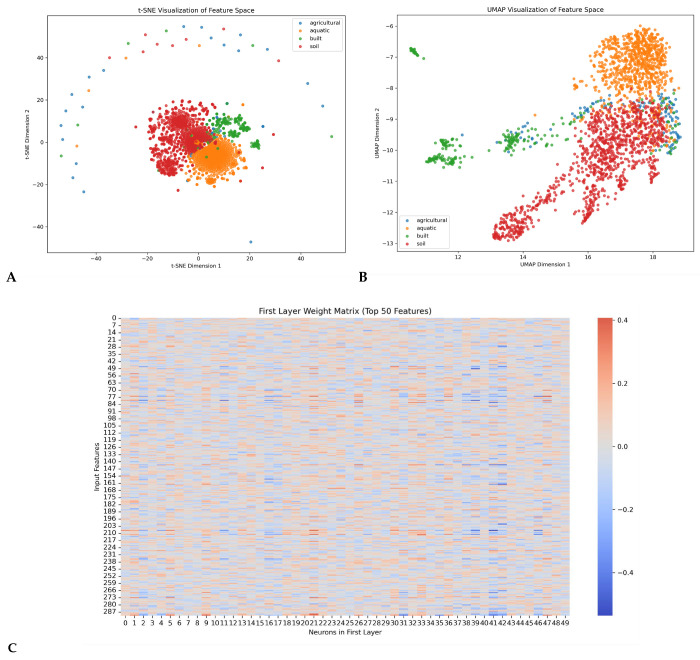
(**A**) t-SNE Visualization of Feature Space Topology—Two-dimensional projection of high-dimensional features using t-distributed Stochastic Neighbor Embedding. Class-specific distributions: Agricultural (blue) exhibits peripheral dispersion, while aquatic (green), built (orange), and soil (red) demonstrate central co-clustering. Partial overlap among non-agricultural classes (PC1 + PC2 = 35.49% variance explained) indicates shared feature subspaces despite categorical distinctions. (**B**) UMAP Embedding of Feature Manifolds—Nonlinear dimensionality reduction via Uniform Manifold Approximation and Projection. Distinct cluster formation observed: Aquatic (orange) forms compact upper-right grouping; soil (red) occupies elongated lower-right structure; built (green) distributes in left-central region; agricultural (blue) shows maximal dispersion. Enhanced spatial segregation versus t-SNE suggests superior discriminative power for peripheral classes. (**C**) First-Layer Feature-Neuron Weight Mapping Weight matrix between input features (*y*-axis, 0–287) and first-layer neurons (*x*-axis, 0–49). Color-mapped weights reveal (i) Strong negative weights (blue, ≤−0.4) concentrated in neurons 25–40, (ii) Positive weights (red, ≤0.2) predominating in early neurons (0–15), and (iii) Global sparsity (white, ≈0) in >65% of connections. Diagonal banding indicates selective feature specialization.

This proof-of-concept study demonstrates that strain-resolved 2bRAD-M profiling combined with a hierarchically attentive neural network enables forensic individual identification with high accuracy under controlled conditions. The distinct clustering of samples from different individuals in the learned feature space, as visualized by t-SNE and UMAP (Figure 5), underscores the model’s ability to capture individual-specific microbial signatures. The HAN model’s superior performance (~98.7% accuracy) over traditional methods highlights the value of attention mechanisms in learning discriminative, individual-specific microbial signatures from complex community data [23,24]. The observed longitudinal stability (ICC = 0.86 over 180 days) challenges assumptions about excessive temporal variability in skin microbiota [40] and suggests that core strains (e.g., *C. avidum*) persist with high host-specificity [41]. The environmental persistence of microbial signatures, with an estimated half-life of approximately 87 days based on our degradation model, significantly exceeds that of touch DNA (half-life ≈ 2.8 days [39]), highlighting a potential advantage for analyzing degraded evidence or samples collected long after deposition.

### 4.1. Clinical and Forensic Implications

The proposed framework shows promise as a complementary tool in forensic investigations, particularly in scenarios where conventional STR profiling fails due to low DNA quantity or quality, or in cases involving mixed samples where the HAN model maintains 87.4% accuracy. Practical integration into existing workflows would require developing standardized sampling kits, establishing consensus on core discriminatory biomarkers, and creating curated reference databases. The model’s ability to incorporate environmental metadata (e.g., particulate matter exposure) could also aid in geolocation or lifestyle inference, adding contextual information to forensic investigations [42,43]. However, its application remains preliminary, and it should currently be viewed as an auxiliary line of evidence rather than a standalone method.

### 4.2. Limitations and Future Directions

A primary limitation of this study is the small size of the core validation cohort (*n* = 6 volunteers), which restricts the generalizability of the high accuracy rates reported. While the use of large public datasets for model development mitigates this to some extent, the findings must be interpreted as proof-of-concept. Future research must prioritize validation in larger, more diverse populations encompassing varied geographies, ancestries, and lifestyles [10,44]. Furthermore, factors such as antibiotic exposure, which reduced discrimination accuracy by 12–18% in our sub-analysis, and hygiene or dietary habits, which were not systematically controlled, represent significant sources of variation that need to be quantified and incorporated into future models, perhaps as covariates [45,46]. The current processing time (~36 h) also exceeds that of rapid STR kits, though parallelization and miniaturization could bridge this gap [47].

The claim of being a “reproducible pipeline” is tempered here to reflect the preliminary nature of the study. Reproducibility across laboratories remains to be demonstrated. Future directions include developing portable sequencing solutions for field deployment [48], establishing international forensic microbiome databases with standardized metadata [49], and integrating multi-omics data (e.g., metabolomics) to enhance discrimination power [50,51]. While the HAN model offers some interpretability through attention weights, future implementations would benefit from advanced explanation techniques (e.g., SHAP values [43]) to meet the stringent transparency requirements of forensic science [52]. Rigorous validation against international standards (e.g., ISFG guidelines [39,53]) is essential before any practical forensic implementation can be considered.

The high LR values reported herein, while demonstrating the potential discriminative power of the framework, are derived from a proof-of-concept validation on a very small cohort (*n* = 6 individuals). Extensive calibration and validation against large, diverse, and population-representative databases are mandatory before such LR estimates can be considered reliable for reporting in actual forensic casework.

While this study accounted for temporal variation within a limited timeframe, the human microbiome is subject to modulation by various extrinsic and intrinsic factors beyond our current experimental design. As noted, antibiotic usage can profoundly alter microbial community structure [54]. Similarly, the consumption of probiotics or fermented foods, which introduce exogenous microbial strains, may cause transient or persistent shifts in an individual’s microbiome profile [55]. Such interventions, along with dietary changes, major illness, or travel, represent potential confounding variables that could affect the stability of an individual’s microbial ‘fingerprint’ over longer periods or under specific circumstances. Future studies with longitudinal monitoring of individuals undergoing such lifestyle or medical interventions are needed to quantify their impact on forensic identification accuracy.

In conclusion, this proof-of-concept study demonstrates the feasibility of using skin and saliva microbiomes for high-resolution individual identification within a forensic framework. The integration of 2bRAD-M sequencing with a HAN model shows promising discriminative power. However, the real-world applicability of this approach will require further validation in larger, more diverse populations and an understanding of how factors like antibiotic use, probiotic intake, and other long-term lifestyle variables influence the persistence of an individual’s microbial signature.

## 5. Conclusions

In summary, this study provides a proof-of-concept that 2bRAD-M microbial fingerprinting integrated with a hierarchical attention network can achieve high individual identification accuracy under controlled conditions. The method demonstrates advantages in resolution and environmental resilience compared to some existing methods. However, the limited validation cohort size underscores the preliminary nature of these results. Substantial further validation is required to overcome limitations related to sample diversity, confounding factors like antibiotic use, and real-world variability before this approach can be seriously considered for routine forensic application.

## Figures and Tables

**Table 1:** Sample Characteristics and Sequencing Statistics.

Sample Type	Number of Samples	Sequencing Depth (M Reads)	Q30 Score (%)	ASVs Identified
Skin Swabs	6	6.8 ± 1.3	93.2 ± 1.5	4523 ± 312
Saliva	6	6.5 ± 1.1	93.7 ± 1.3	5217 ± 287
Surface Transfer	12	5.9 ± 0.9	92.8 ± 1.7	3894 ± 256
Negative Controls	2	0.1 ± 0.05	90.1 ± 2.1	15 ± 8
Total	26	6.7 ± 1.2	93.4 ± 1.8	4798 ± 312

**Table 2:** Microbial Community Composition at the Genus Level.

Genus	Skin Abundance (%)	Saliva Abundance (%)	Feature Importance
*Cutibacterium*	18.7 ± 6.2	1.2 ± 0.4	0.156 ± 0.023
*Staphylococcus*	15.3 ± 4.9	3.8 ± 1.1	0.128 ± 0.019
*Corynebacterium*	11.8 ± 3.7	2.5 ± 0.7	0.094 ± 0.015
*Prevotella*	2.1 ± 0.8	15.6 ± 4.3	0.143 ± 0.021
*Streptococcus*	3.5 ± 1.2	22.3 ± 5.1	0.087 ± 0.014

**Table 3:** Comparative Performance of Different Forensic Identification Methods.

Method	Resolution	Accuracy (%)	LR (Pristine)	Degraded Sample Accuracy (%)	Processing Time (Hours)
2bRAD-M + HAN	Strain-level	98.7	>10^6^	87.4	36
16S rRNA + RF	Species-level	70.2	10^2^–10^3^	45.2	48
16S rRNA + CNN	Species-level	75.8	10^2^–10^4^	52.7	48
STR Profiling	Individual	99.2	>10^6^	25.1	8
mtDNA Sequencing	Haplogroup	85.4	10^1^–10^2^	78.9	24

**Table 4:** Temporal Stability Analysis of Microbial Fingerprints.

Time Point (Days)	Identification Accuracy (%)	ICC Value	Core Taxa Persistence (%)
0	98.7	0.92 ± 0.03	100
30	96.2	0.89 ± 0.04	96.4
60	94.8	0.87 ± 0.05	92.1
90	93.1	0.85 ± 0.05	88.7
120	91.5	0.84 ± 0.06	86.2
150	89.7	0.83 ± 0.06	84.3
180	88.3	0.82 ± 0.07	82.5

## Data Availability

The 2bRAD-M sequencing datasets generated and analysed during the current study are available in the NCBI Sequence Read Archive (SRA) repository, under the BioProject accession number PRJNA1332087. The public datasets from the Qiita database (Studies 797, 1741, 450, 232) analysed during this study are available in the Qiita repository, https://qiita.ucsd.edu/. The original data for these studies are available in the European Nucleotide Archive (Study 797: PRJEB1799; Study 1741: PRJEB5758; Study 232: ERP022626) and from the published article (Study 450: PubMed ID: 19892944, https://doi.org/10.1126/science.1177486).

## Abbreviations

The following abbreviations are used in this manuscript:

2bRAD-M	Type IIB Restriction site-associated DNA Microbiome
AI	Artificial Intelligence
ASV	Amplicon Sequence Variant
AUC	Area Under the Curve
CNN	Convolutional Neural Network
CSS	Cumulative Sum Scaling
DNA	Deoxyribonucleic Acid
GTDB	Genome Taxonomy Database
HAN	Hierarchical Attention Network
HMP	Human Microbiome Project
ICC	Intraclass Correlation Coefficient
LR	Likelihood Ratio
OTU	Operational Taxonomic Unit
PCA	Principal Component Analysis
PERMANOVA	Permutational Multivariate Analysis of Variance
RF	Random Forest
SRA	Sequence Read Archive
STR	Short Tandem Repeat
t-SNE	t-Distributed Stochastic Neighbor Embedding
UMAP	Uniform Manifold Approximation and Projection
